# Comparison of the deep immune profiling of B cell subsets between healthy adults and Sjögren’s syndrome

**DOI:** 10.1080/07853890.2022.2031272

**Published:** 2022-01-31

**Authors:** Ruiling Feng, Jing Zhao, Feng Sun, Miao Miao, Xiaolin Sun, Jing He, Zhanguo Li

**Affiliations:** Department of Rheumatology & Immunology, Peking University People's Hospital, Beijing, China

**Keywords:** B cell subsets, reference ranges, multicolour flow cytometry, healthy adults, Sjögren’s syndrome

## Abstract

**Objectives:**

Detailed analysis targeting B cell subgroups was considered crucial in monitoring autoimmune diseases and treatment responses. Thus, precisely describing the phenotypes of B cell differentiation and their variation in primary Sjögren’s syndrome (pSS) is particularly needed.

**Methods:**

To characterize the proportions and absolute counts of B cell subsets, peripheral blood from 114 healthy adults of China (age range: 19–73 years) and 55 patients with pSS were performed by flow cytometry and CD19, CD20, CD24, CD27, CD38 and IgD were used as surface markers to identify B cell mature process. Age- and gender-stratified analyses were then carried out to improve the interpretation of B cell subsets.

**Results:**

The assessments from healthy adults showed that the proportion of naive B cells presented a significant increase with age. A reversal trend was noted that the percentage of B10 decreased markedly with age. In addition, analysis based on gender showed that the relative percentage and number of naive B cells were higher in females than in males whereas the proportions of switched memory B cells and B10 cells were decreased in female. Patients with pSS exhibited a significant expansion in naïve B cells and unswitched memory B cells, accompanied with decreased switched memory B cells and B10 cells, which were identified to be associated with autoantibody production.

**Conclusions:**

Our study presented a reliable analysis by flow cytometry to cover the principal B cell subtypes. These different stages of B lymphocytes may have implications for evaluating the activation of pSS and other autoimmune diseases and treatment efficacy.

**KEY MESSAGES**
- B cell subsets play a pivotal role in the pathogenesis of primary Sjögren’s syndrome (pSS) and other autoimmune diseases. A practical and accurate flow cytometry method to profile B cell phenotypes in peripheral blood of healthy adults is especially essential.
- Additionally, we presented reliable reference ranges for B cell subsets in regards to the local population. Age- and gender-related analyses are available to better understand their influence in immune status and treatment outcome.
- The distribution of B-cell subsets is found substantially altered in patients with pSS, bringing novel avenues for pSS research in the future.

## Introduction

B cells are multifunctional lymphocytes widely engaging in humoral immunity and immune homeostasis [1]. As the important initiators and effectors of a normal immune response, B cells can activate CD4+ T cells by the presentation of antigens and in turn, be differentiated to express high-affinity antigen-specific antibodies with the help of T follicular helper (TFH) cells [2,3].

With fast development of flow cytometry, the studies of autoimmune diseases and vaccination are mostly dependent on the presence of phenotypic features of B cells in peripheral blood. Simplistically, CD19 + CD20+ B cell development occurs in the bone marrow at the transitional B cell stage where they undergo processes to ensure central tolerance, subsequently, transitional B cells are released into the periphery and migrated to the spleen via the bloodstream to fulfil their maturation into competent naive cells [4,5]. Upon developing into mature B cells by antigen exposure and activation, they recirculate among the lymphoid follicles of spleen and lymph nodes as well as proliferate and differentiate into memory B cells and double-negative (DN) B cells in the presence of T cell help, followed by antibody-producing plasma cells from activated memory B cells in the germinal centre [6,7]. Notably, once emigrated from the bone marrow in humans, B cell subsets can be broadly labelled based on defined surface markers in human peripheral blood such as CD19, CD20, CD24, CD38, IgD and CD27, although the expression levels of surface information varied according to maturation progression [8,9].

Primary Sjögren’s syndrome (pSS) is a chronic autoimmune disorder characterized by lymphocytic infiltration of salivary and lachrymal glands [10]. In light of the pivotal role of B cell subtypes for the pathogenesis of pSS, an improved understanding of B cells in healthy adults is essential in an endeavour to associate the pathogenesis underlying B cells induced pSS with laboratory statistics. Previous reports have suggested that the single platform test using TruCount tubes reduces the difference between inter- and intra-laboratory [11]. However, most of the current researches proposed so far have established the reference ranges by dual-platform method [5,12,13]. Variations in the number and percentage of B cell subsets have been regarded to be influenced by ethnicity, gender, age and environmental factors [14].

Here, to precisely delineate different maturation process of peripheral B cells in healthy adults of China as well as their alterations in patients with pSS, we have defined a seven-colour combination of antibodies covering relatively comprehensive B cell surface information. Then, extensive reference intervals of B cell subsets among Chinese healthy adults have been built. The results assessed and discussed influences of gender and age on lymphocyte composition as well.

## Patients and methods

### Study population and patients

A total of 114 healthy adults of different ages (19–73 years, mean age = 44.9) were enrolled without infection, cancer, immunologic or other chronic disease or ongoing treatment that could affect the immune system. There were 50 males and 64 females in this sample. All the subjects were separated into five age groups and in each group, the ratio of female to male was nearly equal. Additionally, 55 patients with pSS that diagnosed according to the 2016 American College of Rheumatology (ACR)/European League Against Rheumatism (EULAR) criteria, were recruited for further profiling and their routine laboratory results were collected. Sample collection was carried out among nearly one month and any acute infection or drug administration within two weeks before collection was excluded from the group. The study was approved by the Institutional Ethics Committee of Peking University People’s Hospital (2020PHB378-01).

### Sample processing and flow cytometry

Fresh collected whole blood was obtained and processed within 24 h. To determine the proportions of B cell subsets in the periphery, immunophenotyping of B cells was classified using conjugated anti-human murine monoclonal antibodies (mAbs) as follows: anti-CD3-PerCP, anti-CD19-PE-cy7, anti-CD38-allophycocyanin (APC), anti-CD20-APC-CY7, anti-CD24-BV421, anti-CD27-BV510 and anti-IgD-fluorescein isothiocyanate (FITC). All antibodies were purchased from BD Pharmingen (San Diego, CA) and eBioscience (San Diego, CA) (Altrincham, UK). For cell staining on the surface, 100 µL whole blood was incubated with antibodies for 15 min in the dark, at room temperature. Then, 2 mL diluted FACS Lysing Solution (BD Biosciences, San Jose, CA) was added for 10 min of erythrocyte lysis. Thereafter, cells were centrifuged at 600×*g* for 5 min and supernatant was removed. The stained cells were washed in phosphate-buffered saline (PBS), all the samples were kept cool and dark until flow analysis. After proper instrument setting, labelled B cell subsets were acquired on a FACSAria II flow cytometer (BD Biosciences, San Jose, CA) and FlowJov10 (TreeStar, Woodburn, OR) was applied for analysis of each B cell subpopulations.

### Calculation of B cell counts

Furthermore, the absolute counts were detected by using TruCount tubes (BD Biosciences, San Jose, CA). Twenty microlitres of BD multitest CD3/CD45/CD19 was pipetted into the bottom of the tube and mixed with 50 µL anticoagulated whole blood using reverse pipetting technique. Then, the cells were incubated for 15 min in the dark at room temperature followed by 450 µL BD FACS lysing solution added. All the samples were stored and ready to be analysed on the flow cytometer.

### Statistical analysis

After the compensation matrix was adjusted, over 10 samples were concatenated and analysed using FlowJov10 (TreeStar, Woodburn, OR) plugins including down-samples and t-SNE. Data were analysed using SPSS Statistics 24.0 software (SPSS Inc., Armonk, NY). Data were reported as mean, median, standard deviation (SD) and quartile range. After the verification of the normal distribution of the data, gender differences and the comparison between healthy individuals and the pSS patients were examined for statistical significance using Mann–Whitney’s test. For multiple group comparisons distributed by age, the Kruskal–Wallis test was performed and correlation coefficients were determined by simple linear regression. A *p* value lower than .05 denoted a statistically significant difference. The outlier values in the study were investigated and included in the analysis if there were no valid reasons for exclusion.

## Results

Our study enrolled a total of 55 patients with pSS (20–69 years, mean age = 49.2). The characteristics of pSS patients and healthy adults were demonstrated in Table 1. The healthy group consisted of 114 Chinese people with the age ranging from the young to the elderly, including 50 males and 64 females. As shown in Table 2, we assigned the volunteers to five age groups: 18–30 years (*n* = 22), 31–40 years (*n* = 23), 41–50 years (*n* = 21), 51–60 years (*n* = 31), 61–75 years (*n* = 17) and demographic details of the tested population were presented. The absolute numbers and proportions of distinct B cell subtypes are presented in Table 3. All the data were expressed as mean ± SDs. The 2.5 percentile and 97.5 percentile of the study population, supplemented with the 95% confidence interval were calculated to determine the reference intervals of B cell populations.

**Table 1.** The characteristic of the pSS patients and healthy adults.

	pSS (*n* = 55)	Healthy adults (*n* = 114)
Age, years, mean ± SD	49.19 ± 12.38	44.9 ± 13.96
Female/male	55/0	64/50
ESSDAI, mean ± SD	3.94 ± 1.13	NA
Diseases duration years (range)	3 (2–7)	NA
ESR (mm/h)	33 ± 18	NA
Anti-SSA positivity, *n* (%)	46/50 (92%)	NA
Anti-SSB positivity, *n* (%)	24/50 (48%)	NA
α-Fod, range	14.63 (10.06–25.86)	NA
RF titre, IU/mL, range	159 (55.4–392.0)	NA
IgA titre, g/L, mean ± SD	4.14 ± 1.83	NA
IgG titre, g/L, mean ± SD	24.44 ± 5.60	NA
IgM titre, g/L, mean ± SD	1.46 ± 1.20	NA
C3, g/L, mean ± SD	1.007 ± 0.214	NA
C4, g/L, mean ± SD	0.193 ± 0.062	NA

NA: no data available; pSS: primary Sjögren’s syndrome; ESSDAI: EULAR Sjögren’s Syndrome Disease Activity Index; ESR: erythrocyte sedimentation rate; α-Fod: alpha-fodrin; RF: rheumatoid factor; Ig: immunoglobulin.

The information was collected and showed as mean ± SD or median (25th–75th percentiles).

**Table 2.** Demographics of the population under study.

Age	Mean ± SD	Total	Gender ratio (F/M)
18–30	25.05 ± 3.54	22	12/10
31–40	34.57 ± 2.97	23	11/12
41–50	45.62 ± 3.07	21	12/9
51–60	55.58 ± 2.71	31	20/11
61–75	64.18 ± 3.15	17	9/8

The subjects were averagely grouped by age including people aged 18–30, 31–40, 41–50, 51–60 and 61–75. Additionally, the ratio of female to male is relatively equal in our study. Unswitched memory B cells: CD19 + IgD + CD27+ B cells; switched memory B cells: CD19 + IgD–CD27+ B cells; DN B cells: double negative B cells; B10 cells: CD19 + CD24hiCD27+ B cells.

**Table 3.** Normal reference ranges for absolute numbers and proportions of various B lymphocyte subset in healthy adults.

	Mean ± SD	Median	Quartile range	CI^a^	Reference range^b^
B cell %	12.61 ± 4.45	12.50	5.58	11.78–13.43	5.17–23.60
B10 cell %	25.35 ± 10.05	25.20	15.13	23.49–27.22	8.99–48.43
DN B cell %	7.87 ± 6.17	6.38	5.31	6.72–9.01	2.35–22.79
Naive B cell %	60.14 ± 12.25	60.65	15.50	57.87–62.41	31.45–81.69
CD19 + CD20+ B cell %	98.29 ± 1.40	98.80	1.50	98.03–98.55	94.00–99.70
CD19 + CD20– B cell %	0.81 ± 0.84	0.58	0.58	0.66–0.97	0.12–3.72
Plasma cell %	0.44 ± 0.67	0.25	0.32	0.32–0.57	0.02–2.95
Unswitched memory B cell %	10.82 ± 7.14	9.47	8.38	9.49–12.14	1.44–28.34
Switched memory B cell %	21.18 ± 8.05	20.30	10.53	19.68–22.67	7.51–43.04
B cell numbers (cells/µL)	280.04 ± 148.64	247.00	131.00	252.46–307.62	86.30–604.63
B10 cell numbers (cells/µL)	68.9 ± 40.41	59.02	51.16	61.40–76.40	13.11–186.40
DN B cell numbers (cells/µL)	21.83 ± 23.86	16.91	14.28	17.41–26.26	3.70–126.84
Naive B cell numbers (cells/µL)	171.21 ± 108.36	146.76	102.46	151.10–191.31	38.95–442.83
CD19 + CD20+ B cell numbers (cells/µL)	274.98 ± 144.17	240.94	129.50	248.23–301.74	85.75–594.53
CD19 + CD20– B cell numbers (cells/µL)	2.29 ± 2.54	1.39	2.33	1.82–2.76	0.30–10.76
Plasma cell numbers (cells/µL)	2.26 ± 2.55	1.39	2.30	1.78–2.73	0.27–10.76
Unswitched memory B cell numbers (cells/µL)	29.40 ± 23.53	24.69	26.73	25.04–33.77	2.95–108.13
Switched memory B cell numbers (cells/µL)	57.62 ± 33.27	49.25	40.68	51.45–63.79	8.76–154.97

Analysis of B cell and its subsets in all enrolled healthy people. All the data from 114 healthy adults were demonstrated as mean ± SD, median, quartile range complemented with reference range and confidence interval. Unswitched memory B cells: CD19 + IgD + CD27+ B cells; switched memory B cells: CD19 + IgD–CD27+ B cells; DN B cells: double negative B cells; B10 cells: CD19 + CD24hiCD27+ B cells.

^a^ 95% confidence interval of the sample mean.

^b^ 2.5–97.5th percentile of reference range.

### Identification of B lymphocyte subset by flow cytometry

All the lymphocyte population was detected on the basis of forward scatter (FSC) and side scatter (SSC) characteristics. Total B cells were identified by flow cytometric analysis of the lineage markers CD3– and CD19+. Afterwards, we also evaluated the different maturation stages and phenotypic features of B cells similarly. On average, CD19 + CD20+ and CD19 + CD20– were gated for further identification. The presence of IgD and CD27 was used to discriminate naive (CD19 + IgD + CD27–) B cells from unswitched memory (CD19 + IgD + CD27+) B cells and switched memory (CD19 + IgD–CD27+) B cells. Conversely, DN B cells were identified with loss of IgD and CD27. Except for CD27 expressed, additional staining of CD24hi was selected to define B10 (CD19 + CD24hiCD27+) cells and CD38hi was defined as plasma cells (CD19 + CD20–CD27 + CD38hi) (Figure 1(A)). In terms of absolute numbers, lymphocytes were gated by CD45/side scatter dot plots along with B cells were defined as CD3–CD19+ lymphocytes (Figure 1(B)). Besides, the counts of each subtype were enumerated according to their percentages of total B cells.

Figure 1.Design of human B cell-focused flow cytometry panel. (A) Sequential gating strategy for identification of B cells and downstream analysis. After lymphocyte gating according to FSC and SSC parameters, CD19-positive lymphocytes were selected and further resolved into distinct subpopulations via the analysis of CD20, CD24, CD27, CD38 or IgD expression. (B) Calculation of absolute numbers. Lymphocytes were gated according to CD45/side scatter dot plots. Then, total B cells were defined as CD3–CD19+ lymphocytes. (C) t-SNE analysis objectively delineates B cell subsets in peripheral blood of healthy individuals. tSNE-based dimension reduction was performed on B cells revealing distinct, shared and diverse lymphocyte phenotypes. Unswitched memory B cells: CD19 + IgD + CD27+ B cells; switched memory B cells: CD19 + IgD–CD27+ B cells; DN B cells: double negative B cells; B10 cells: CD19 + CD24hiCD27+ B cells.

**Figure:**
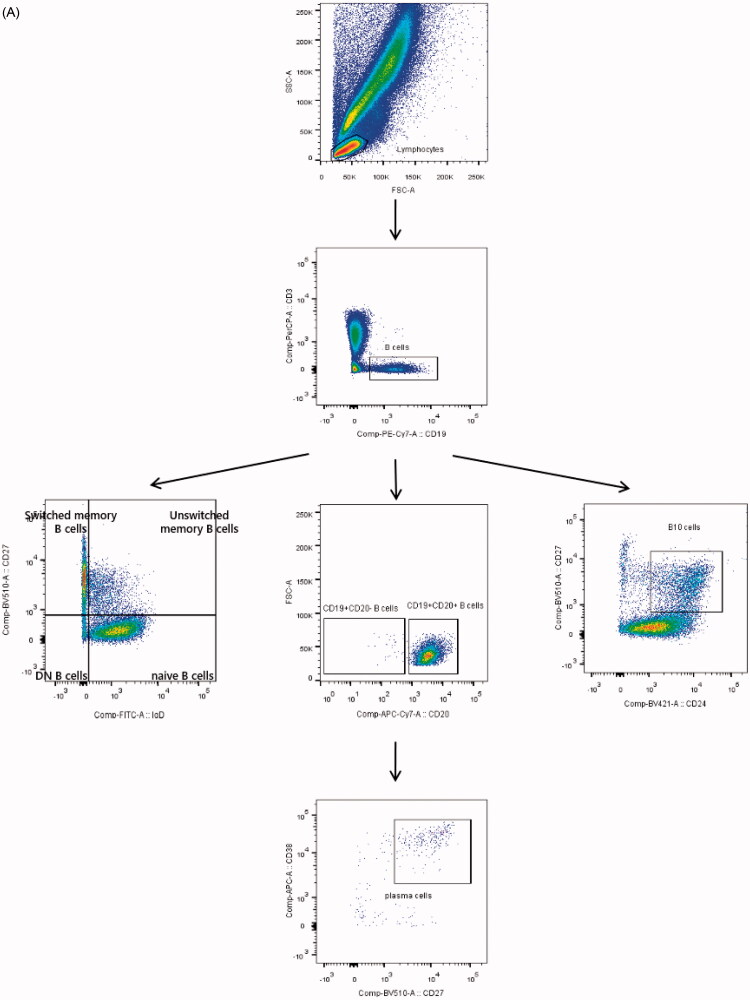

**Figure:**
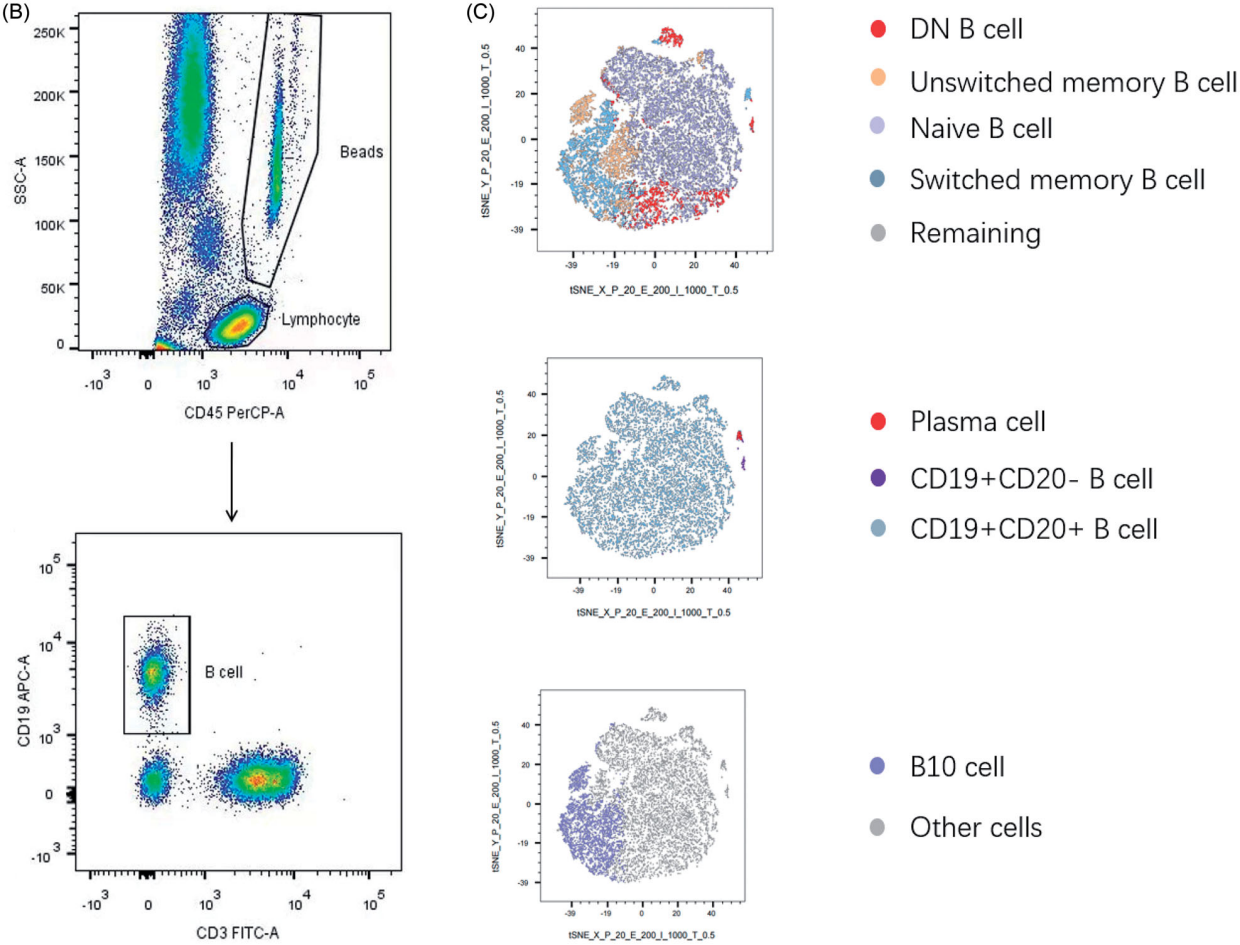

### Distinct B cell clusters separated by t-SNE

In an attempt to visualize multiparameter flow cytometric measurements, the techniques of clustering and dimensionality reduction are definitely required to segregate cell populations automatically while reserving useful features of each data [15]. Currently, t-SNE is the most commonly used tool in single-cell analysis, more than 10 collections were included to check sample quality and B cells from individual samples were down-sampled to 5000 events per sample, and then concatenated for downstream analyses. The t-SNE outcomes showed the coherent populations with phenotypes displayed by flow cytometry with seven-colour panel, suggesting stable and reasonable staining protocols of B cells in our study (Figure 1(C)).

### Age specific reference values in healthy adults

**Figure 2.**
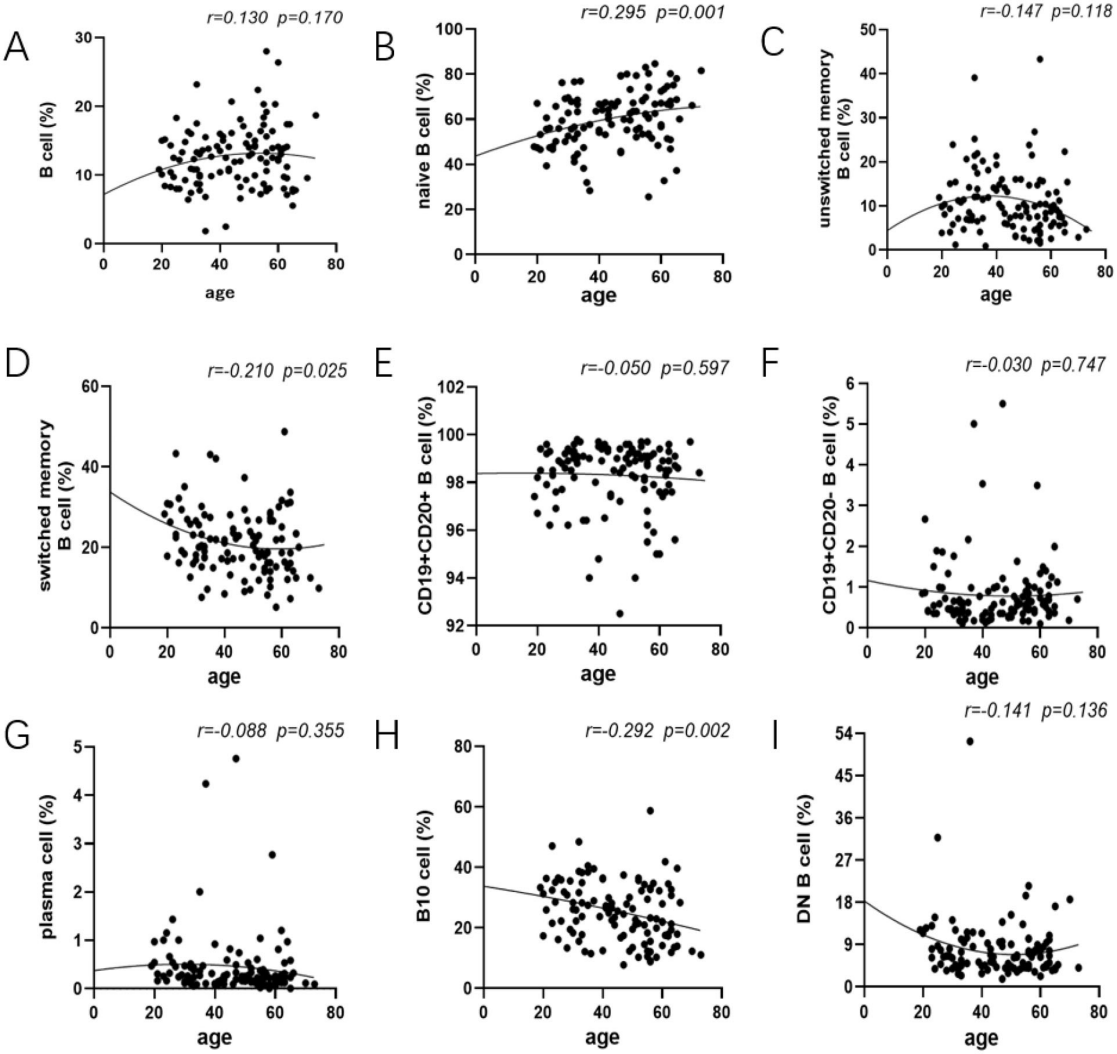
Changes of the frequency of B cell subsets at each age state. The healthy donors in our study aged between 19 and 73, and they were divided into five groups at nearly 10 years intervals. Peripheral blood was collected and prepared to analyse mounting parameters by flow cytometry. In addition, a Pearson correlation was performed between B cell subsets and age and linear regression was used to plot graph. (A) No substantial difference was noted in total B cell of each group. (B, D, H) The prevalence of naive B cells showed a marked rise whereas IgD–CD27+ B cells and B10 cell encompassed the declined tendency with statistical significance. (C, N) Decreasing proportions of IgD + CD27+ B cells and DN B cell were noted as age increased with no significant difference. (E, F, G) Comparison of CD19 + CD20+ B cells along with CD19 + CD20– B cells, plasma cells, exhibited no significant effect between age groups. Unswitched memory B cells: CD19 + IgD + CD27+ B cells; switched memory B cells: CD19 + IgD–CD27+ B cells; DN B cells: double negative B cells; B10 cells: CD19 + CD24hiCD27+ B cells. Data were analysed with non-parametric Mann–Whitney’s test or one-way analysis of variance (ANOVA) with *p*<.05 indicating a significant difference. Pearson’s *r* values for the linear curves expressing the correlation between age and different subtypes.

To analyse the changes of B cell subpopulations related to ages, peripheral blood samples were drawn from healthy adults of various age groups performed by flow cytometry. Data showed that there was no significant difference among those five groups of total B cells (proportion: *r* = 0.130, *p*=.170; counts: *r* = 0.095, *p*=.314; Figure 2(A), Figure S1A). The age-related changes were expressed that the proportions and absolute counts of naive B cell were considered to go up with increasing age (proportion: *r* = 0.295, *p*=.001; counts: *r* = 0.187, *p*=.046; Figure 2(B), Figure S1B). In contrast, our analysis demonstrated that the proportions of unswitched memory B cells reduced strikingly, especially in individuals ≥40 years old and DN B cells had a tendency to decline with age although both did not reach significance (unswitched memory B cells: *r*=–0.147, *p*=.118; DN B cells: *r*=–0.141, *p*=.136; Figure 2(C,I)). Besides, the percentage of switched memory B cells and B10 cells experienced a striking decrease with age (switched memory B cells: *r*=–0.210, *p*=.025; B10 cells: *r*=–0.292, *p*=.002; Figure 2(D,H)). These differences throughout life highlights the importance of creating age specific reference patterns. Furthermore, we also observed that the percentage of CD19 + CD20+ B cells together with CD19 + CD20– B cells, plasma cells, exhibited a decrease but not significant effect by age among the groups (CD19 + CD20+ B cells: *r*=–0.050; CD19 + CD20– B cells: *r*=–0.030; plasma cells: *r*=–0.088; *p*>.05; Figure 2(E,F,G)).

### Gender specific reference values in healthy adults

**Figure 3.**
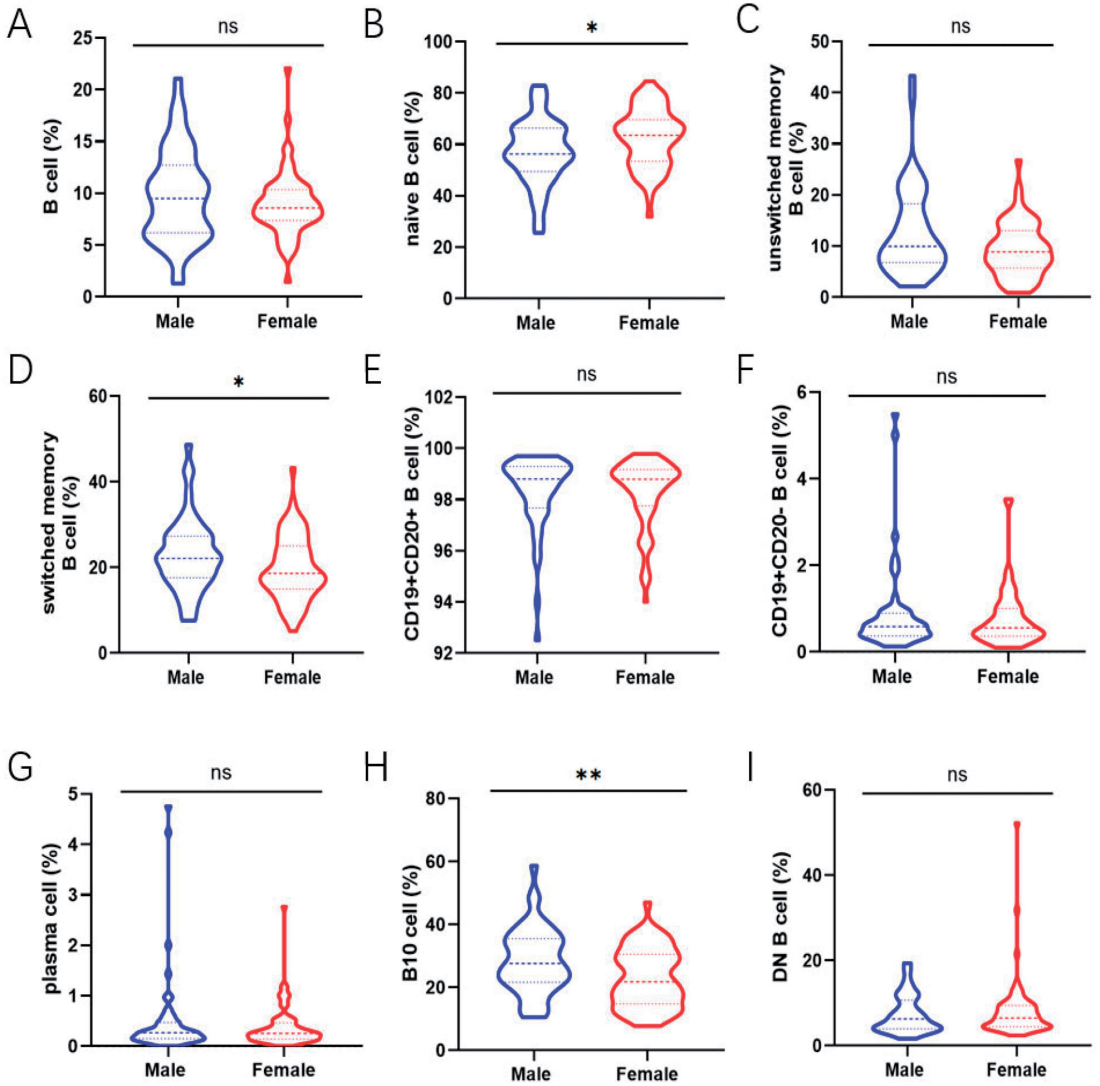
Comparison of the frequency of B-cell subsets between male and female. Relative frequencies of different B cell subsets in each individual were shown by violin plot according to gender. (A, C, E, F, G, I) No gender-dependent difference was noted in total B cell, unswitched memory B cells, CD19 + CD20+ B cell, CD19 + CD20– B cell, plasma cell and DN B cell. (B) The prevalence of naive B cell in female were expressed higher than that in male (*p* = .013). (D, H) The proportion of switched memory B cells and B10 cells in male was expanded compared with that in female (switched memory B cells: *p* = .038; B10 cell: *p* = .004). Data were analysed with non-parametric Mann–Whitney’s test or Student’s *t*-test with *p* < .05 indicating a significant difference. *p < .05; **p < .01; unswitched memory B cells: CD19 + IgD + CD27+ B cells; switched memory B cells: CD19 + IgD–CD27+ B cells; DN B cells: double negative B cells; B10 cells: CD19 + CD24hiCD27+ B cells.

Gender-related changes in total B cells as well as disparate B cell subsets are shown to elucidate how sex hormone exerted on lymphocytes. The percentage of naive B cells in male and female was 56.95 ± 12.72% and 62.64 ± 11.34% followed by switched memory B cells being 22.95 ± 8.51 and 19.80 ± 7.45% respectively. This showed a trend to have an obvious difference between them (naive B cells: *p*=.013; switched memory B cells: *p*=.038; Figure 3(B,D)). Expanding proportions and absolute counts of B10 cells in male were also observed compared to that in female (proportion: *p*=.004; counts: *p*=.015; Figure 3(H), Figure S2H). In addition, the frequency of total B cells and other B cell subsets encompassed no apparent gender-dependent differences (Figure 3).

### B cell subset profiling in Sjögren’s syndrome

**Figure 4.**
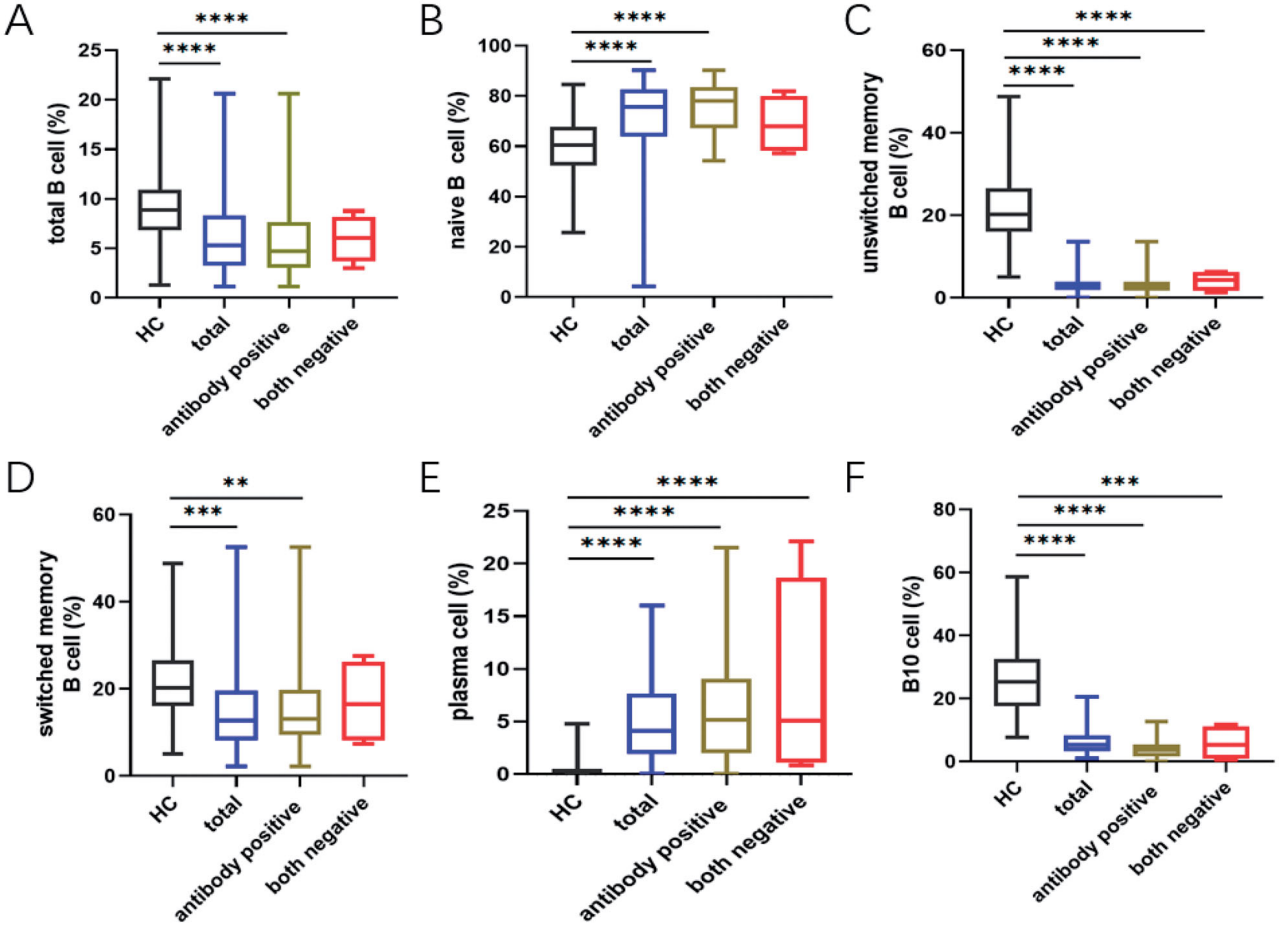
The presence of anti-SSA or SSB exacerbate the abnormal distribution of different B cell subsets. (A) Total B cells, (B) CD19 + CD24 + CD27+ B10 cells, (C) CD19 + CD27-IgD + naïve B cells, (D) CD19 + CD27 + CD38+ plasma cells, (E) IgD + CD27+ unswitched memory B cells and (F) IgD–CD27+ switched memory B cells in pSS patients without both anti-SSA and SSB, as well as patients with one of the antibodies positive compared to the healthy group. Statistically significant differences are indicated by **p < .01; ***p < .001; ****p < .0001. HC: healthy control.

**Figure 5.**
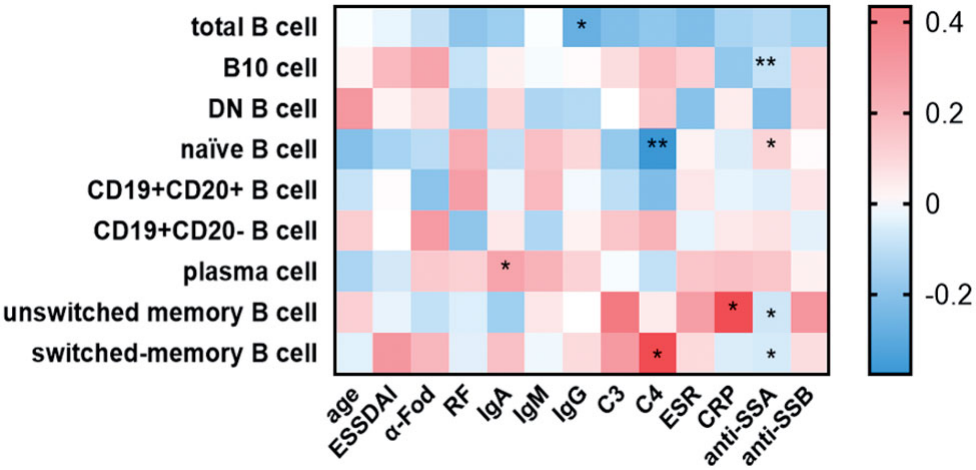
The associations between different B cell subgroups and laboratory data from the pSS patients. Frequencies of certain B cell subsets of pSS patients were acquired, and their correlation analysis with clinical and serological parameters was performed. Data were analysed with Pearson’s and Spearman’s correlation test. *p<.05; **p<.01; unswitched memory B cells: CD19 + IgD + CD27+ B cells; switched memory B cells: CD19 + IgD–CD27+ B cells; DN B cells: double negative B cells; B10 cells: CD19 + CD24hiCD27+ B cells.

**Figure 6.**
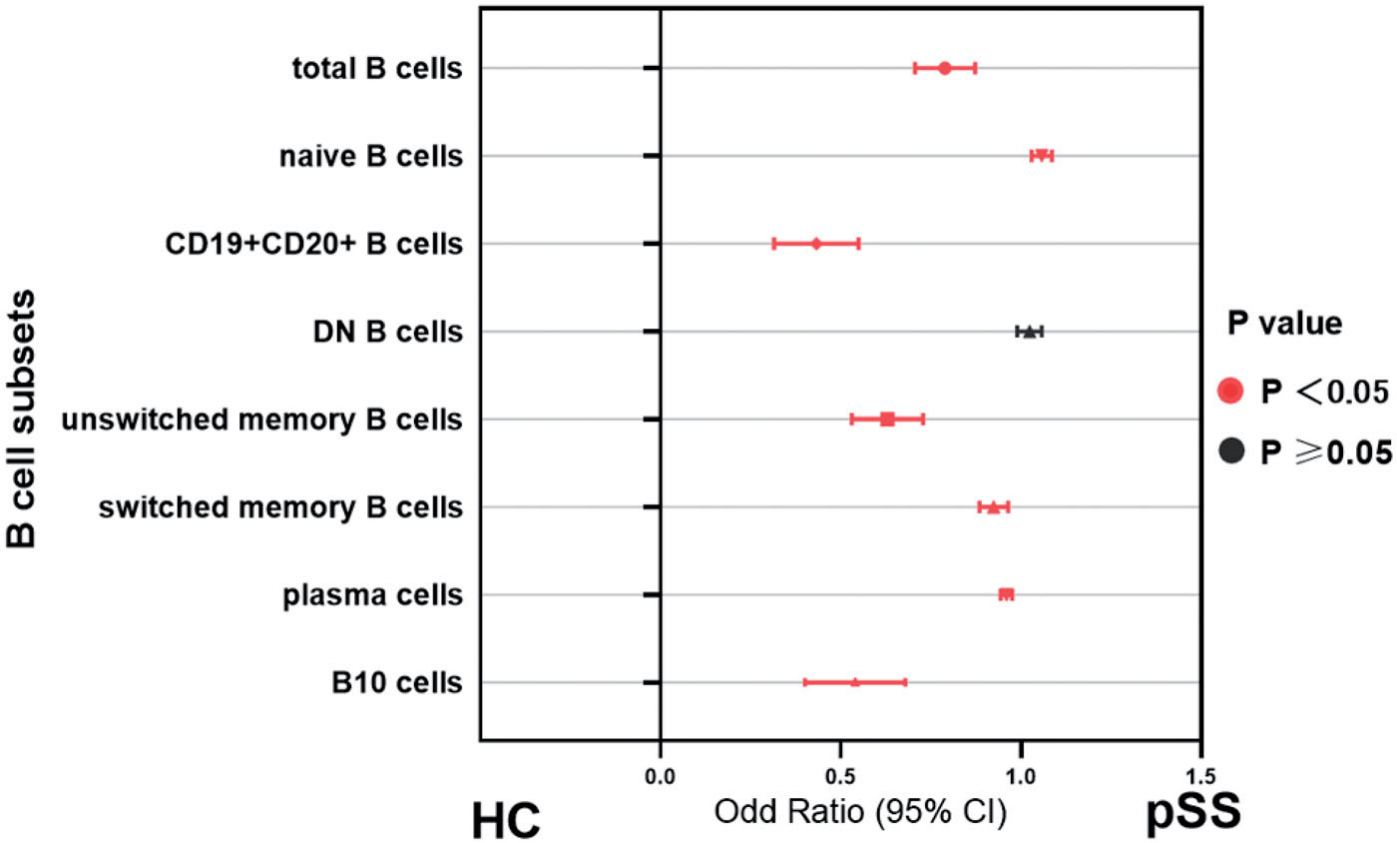
Disrupted B-cell subsets in patients with pSS compared to healthy individuals. The association between the immunophenotypes of B cells and patient groups was assessed by univariate logistic regression analysis. Odds ratios (ORs) and the 95% confidence interval (CI) were computed as well as *p* values. Nearly, all the parameters except DN B cells embraced statistical significance in pSS patients. HC: healthy control; unswitched memory B cells: CD19 + IgD + CD27+ B cells; switched memory B cells: CD19 + IgD–CD27+ B cells; DN B cells: double negative B cells; B10 cells: CD19 + CD24hiCD27+ B cells.

As expected, patients with pSS had disturbed B cell profiles compared to healthy adults. In parallel with the age-specific changes in the healthy group, the percentage of naïve B cells was significantly increased whereas B10 cells reached the bottom in patients between 40 and 50 years (Figure S4). In order to investigate whether B cell changes were correlated with serological findings, autoantibody levels were analysed to classify the patients. Data showed that naïve B cells were substantially developed in the total patients group (*p*<.0001), and patients with anti-SSA or SSB positive showed higher percentages than those with both negative (HC vs. antibody positive: *p*<.0001; HC vs. both negative: *p*=.1692; Figures S3B and 4B). Besides, the prevalence of unswitched memory B cell tended to drop dramatically in pSS patients compared to healthy adults (Figures S3C and 4C). Moreover, the antibody positive group presented the significantly reduced ratio in switched memory B cells (*p*=.0019), as compared with both negative patients (*p*=.3082, Figures S3D and 4D). Simultaneously, the frequencies of CD19 + CD20+ and CD19 + CD20– B cells were also dramatically changed in pSS patients (Figure S3E and F). After differentiation towards plasma cells mostly, peripheral plasma cells were elevated significantly in the whole pSS patients (*p*<.0001, Figure 4(E) , Figure S3G). As B10 cells were found markedly lower in pSS patients than healthy group, the difference presented more pronounced in antibody positive patients (HC vs. antibody positive: *p*<.0001; HC vs. both negative: *p*=.0002; Figure 4(F), Figure S3I). However, total B cells were reduced in pSS patients which might be resulted from the strong treatment (Figure 4(A), Figure S3A). There was no statistical significance of DN B cells in our study (Figure S3I). Further, we analysed the correlation between B cell subtypes and laboratory parameters. As expected, elevated serum IgA was observed positively related to plasma cells (*r* = 0.177, *p*=.021) followed by a significant negative correlation between the levels of IgG and total B cells (*r*=–0.278, *p*=.04). The diminished C4 levels revealed a negative association with the prevalence of naïve B cells, contrary to the involvement with switched-memory B cells (naïve B cell: *r*=–0.376, *p* = 0.005; switched memory B cell: *r* = 0.33, *p*=.015; Figure 5). Moreover, univariate logistic regression analysis shared the similar outcomes that nearly all the B cell subpopulations except DN B cells participated in pSS pathogenesis (Figure 6). Thus, these comprehensive comparison analyses suggest that B cells are closely associated with pSS patients and the further exploration of B cells is notably indispensable in clinical practice.

## Discussion

Flow cytometry emerged as a widely adopted tool for the diagnosis of autoimmune diseases and evaluation of vaccination effectiveness, is available for quantification of lymphocytes and supervision of immune cell dynamics conveniently [16]. Given that loss of immune tolerance and pathogenic interactions between immunological components such as B cells and effector cells, engage in pSS [17,18], the availability of reference intervals with the emphasis on autoimmune-associated B cells occupies an imperative position on facilitating the objective interpretation of abnormal results and managing follow-up patients. Considering genetics, gender, age and environmental factors are indispensable to determine the number and percentage of B cell subsets [19], we therefore established a more reliable reference range for principal B lymphocyte subsets involving healthy adults in particular region and variations in genetics and environmental factors were addressed briefly.

In regard to age-stratified study, no difference of total B cells among those five groups was found, remaining relatively stable in adulthood as previously described [20], but this seems different from Lin et al.’s study which suggests that total B cells drop with age [21]. This may be explained that our volunteers were apparently young, since B cells decline extremely after the age of 80 [22]. In contrast, we observed that naive B cells tended to develop with increasing age consistent with data already published, probably because naive B cells have become gradually resistant to apoptosis with age [23–26]. Interestingly, our finding is not in agreement with the former data reported by Bulati et al. [27], as they showed a decrease of naive B cells in the elderly, it may account for the fact that advancing age is correlated with decreased production of high-affinity antibodies against foreign antigens [28,29]. Further investigations are urgently required to illuminate this discrepancy of naive B cells and the underlying mechanism. Notably, in addition to the markedly reduced of plasma cells, we also found comparable results with former researches that the percentages of switched memory B cells diminished with age [24,30], despite without any significant difference in our research which possibly requires expanding samples. Furthermore, our findings showed striking decreases of unswitched memory B cells and B10 cells with age. It is assumed that due to the altered function of ageing immune system and changes in antibody affinity, a decrease in both of two CD27+ memory B cell pools is accompanied by impaired differentiation into plasma cells [25]. Additionally, a defect in B10 cells can also result in a significant decline in induction of IL10 expression with age due to an elevation in serum autoantibody of healthy older individuals including rheumatoid factors (RFs) [26]. These differences throughout life provide evidence that human ageing could affect peripheral B cell development and increase the incidence of autoimmune diseases [31]. Therefore, establishing age specific reference ranges is utmostly valuable for future exploration of immune system disorders. However, we also discovered that during age, the percentage and number of DN B cells, companied with CD19 + CD20+ B cells, CD19 + CD20– B cells showed no statistical effect.

The influence of gender on B lymphocyte subgroups in healthy adults was also investigated in our study. As results indicated that naive B cells were obviously higher in the female population than male, lower proportion of switched memory B cells and B10 cells were seen in female compared to male. One possible explanation for the gender difference may be associated with comparatively activated immune system in the young female [32]. Numerous lines of evidence suggested that elevation in oestrogen and prolactin can enhance the survival, maturation and regulation of high affinity autoreactive B cells [33]. Of note, oestrogen also negatively regulated B cell lymphopoiesis, can contribute to reduced B lymphocytes, which in turn increase the production of naive B cells in order to maintain B cell immune balance [34,35].

Collectively, our study supported that people around 40 especially women are more susceptible to pSS which may attribute to a pile of naïve B cells and absent B10 cells. Thus, the analysis about age and gender on B cell subsets in healthy adults has a paramount role to play in further exploring the alterations between healthy adults versus pSS patients. Typically, pSS patients show a large predominance of circulating naive B cells suggesting the impaired early B cell tolerance checkpoints in pSS [36]. In addition, there was a reduction in CD20+ B cells and a significant increase of plasma cells in the peripheral blood. Bharaj et al. reported that the accumulation of CD20+ B cells and a decline in CD138+ plasma cells reflected the high inflammatory disease severity in the minor salivary gland (MSG) tissue as they may be recruited into glandular tissues as inflammation developed [37]. We found that memory B cells including unswitched and switched memory B cells experienced a considerable reduction in peripheral blood, reflecting their likely accumulation in exocrine glands and skins of pSS patients and a probable differentiating towards plasma cells as described by Cornec et al. and Ibrahem [38,39]. Considering the reduced proportion of B10 cells followed by reduced secretion of IL10, their failure to restrain type I interferons coupled with the hyperactivation of plasmacytoid dendritic cells (pDCs) might engage in the pathogenesis of pSS [40].

To be mentioned, there inevitably existed slight alterations in different studies about the enumeration and ranges reported for the B cell subsets, probably as a consequence of various factors such as different sample size, uncontrollable methods and lack of standardize results analyses. Meanwhile, our research did have certain limitations that first, a larger cohort of adults, particularly the elderly, is warranted to confirm a normal reference values of B cell subsets for error reduction on the basis of ethnicity, gender, age and environmental factors. Then, we collected the data from lymphocyte subsets of peripheral blood while those of lymphoid tissues were not taken into account in this study. However, as our seven-colour combination of antibodies have been applied to screen immune status, we have established a relatively reliable reference range for B cell subsets of local healthy adults dependent on age and gender. This can advance the general concept of immune disorders and vaccine responses and interpret the results of lymphocyte immunophenotyping logically. Characterizing human B cell subsets would be of great benefit to optimize the therapy of pSS and evaluate their efficacy in clinical application.

## Conclusions

We provided a reliable flow cytometry analysis to establish reference intervals among healthy individuals for better interpreting the disease activation of pSS and treatment efficacy in clinical practice. Age- and gender-related partitions help to achieve a more accurate and reliable appreciation of the changes in B cells. The alterations occurring in pSS patients can therefore bring new perspectives for pSS and other autoimmune diseases in the future.

## Supplementary Material

Supplemental Material

## Data Availability

The datasets during the current study are available from the corresponding author on reasonable request.
