# Supplementary material for: Comparison of the deep immune profiling of B cell subsets between healthy adults and Sjögren’s syndrome
Source: Ann Med. 2022 Jan 31;54(1):472–83. doi: 10.1080/07853890.2022.2031272 (PMC8812739; doi:10.1080/07853890.2022.2031272)
Supplement: Supplemental Material [file IANN_A_2031272_SM5546.pptx]

## Slide 1
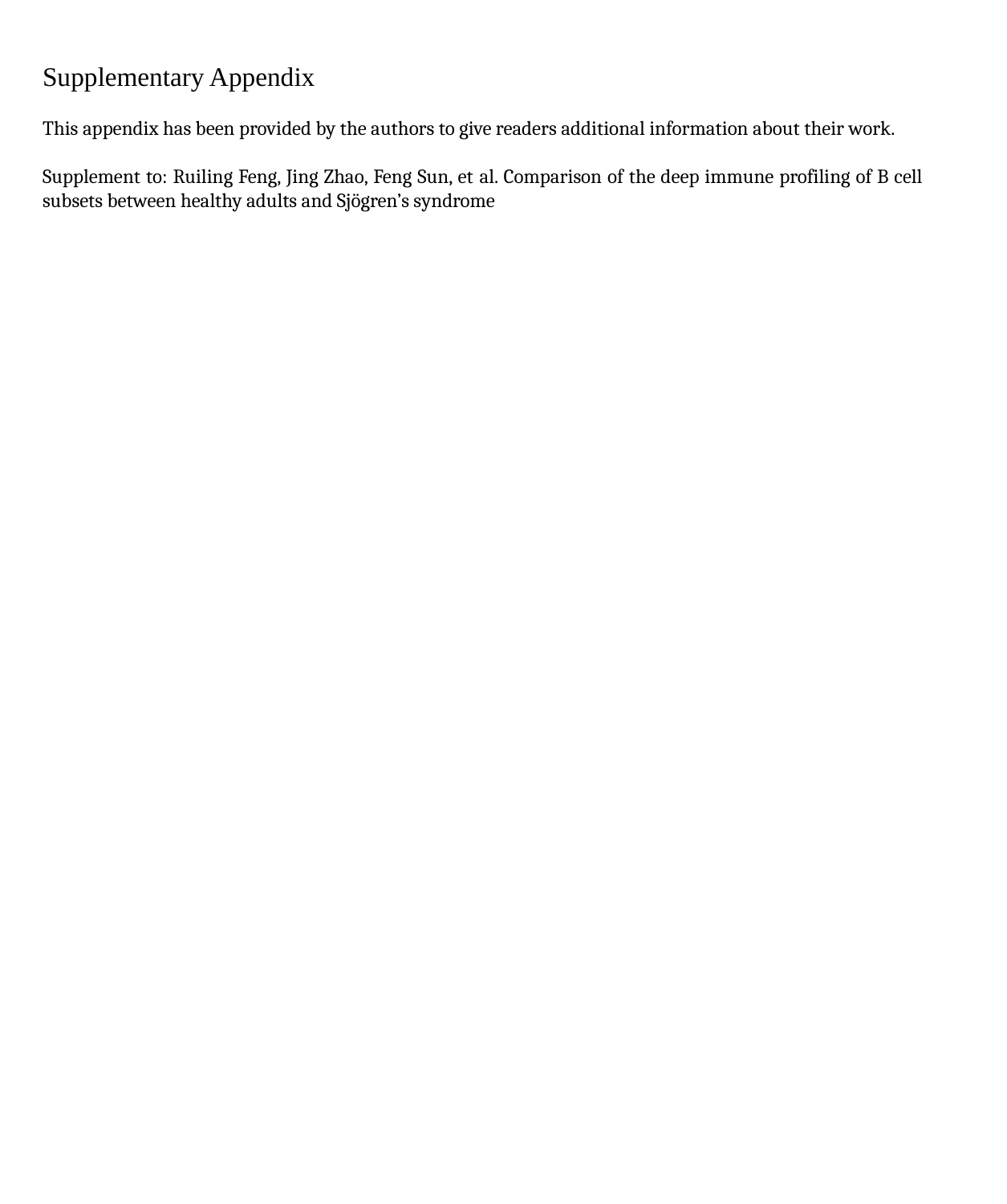

Supplementary Appendix
This appendix has been provided by the authors to give readers additional information about their work.
Supplement to: Ruiling Feng, Jing Zhao, Feng Sun, et al. Comparison of the deep immune profiling of B cell subsets between healthy adults and Sjögren’s syndrome

## Slide 2
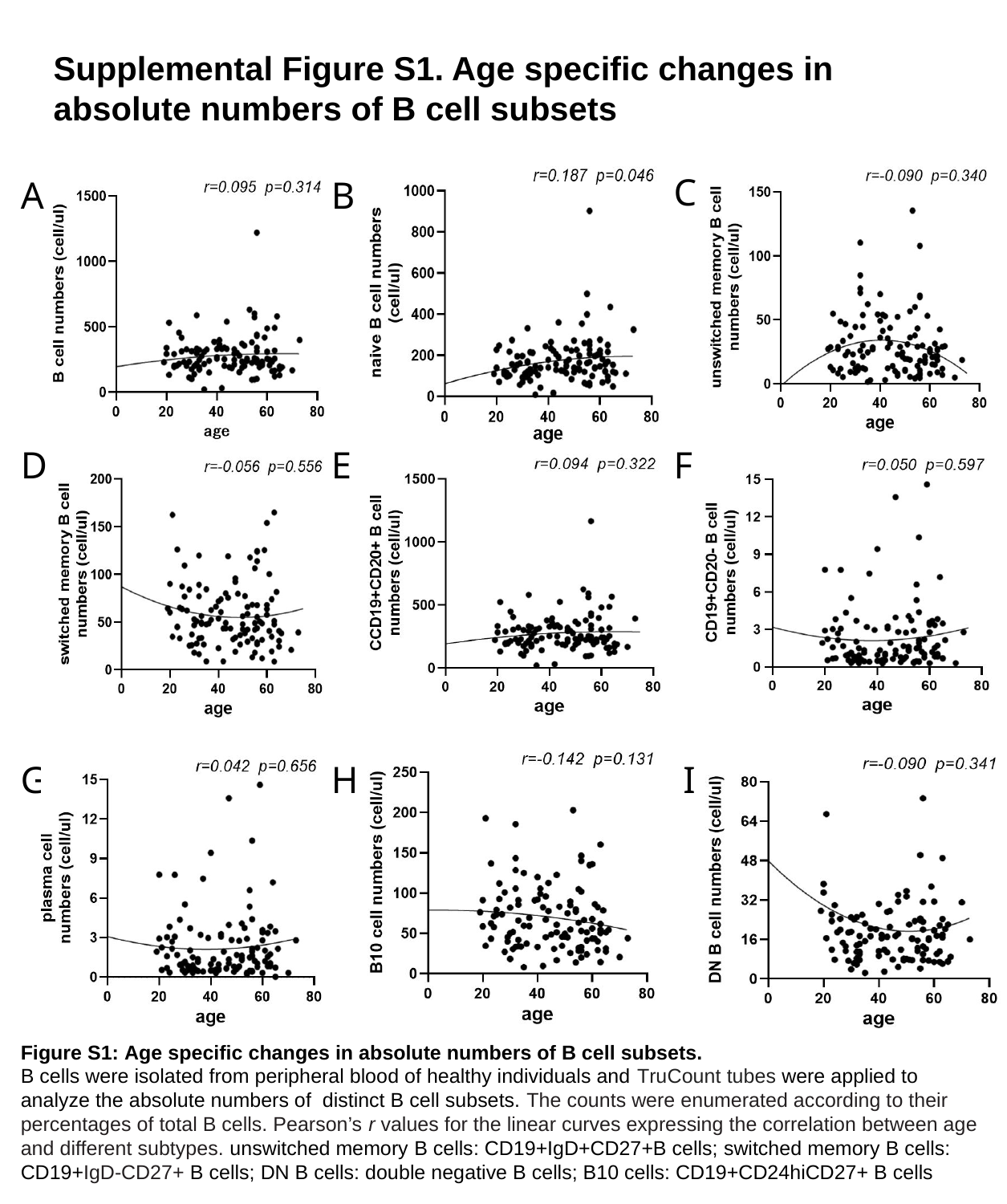

Supplemental Figure S1. Age specific changes in absolute numbers of B cell subsets
C
A
B
F
E
D
G
H
I
Figure S1: Age specific changes in absolute numbers of B cell subsets.
B cells were isolated from peripheral blood of healthy individuals and TruCount tubes were applied to analyze the absolute numbers of distinct B cell subsets. The counts were enumerated according to their percentages of total B cells. Pearson’s r values for the linear curves expressing the correlation between age and different subtypes. unswitched memory B cells: CD19+IgD+CD27+B cells; switched memory B cells: CD19+IgD-CD27+ B cells; DN B cells: double negative B cells; B10 cells: CD19+CD24hiCD27+ B cells

## Slide 3
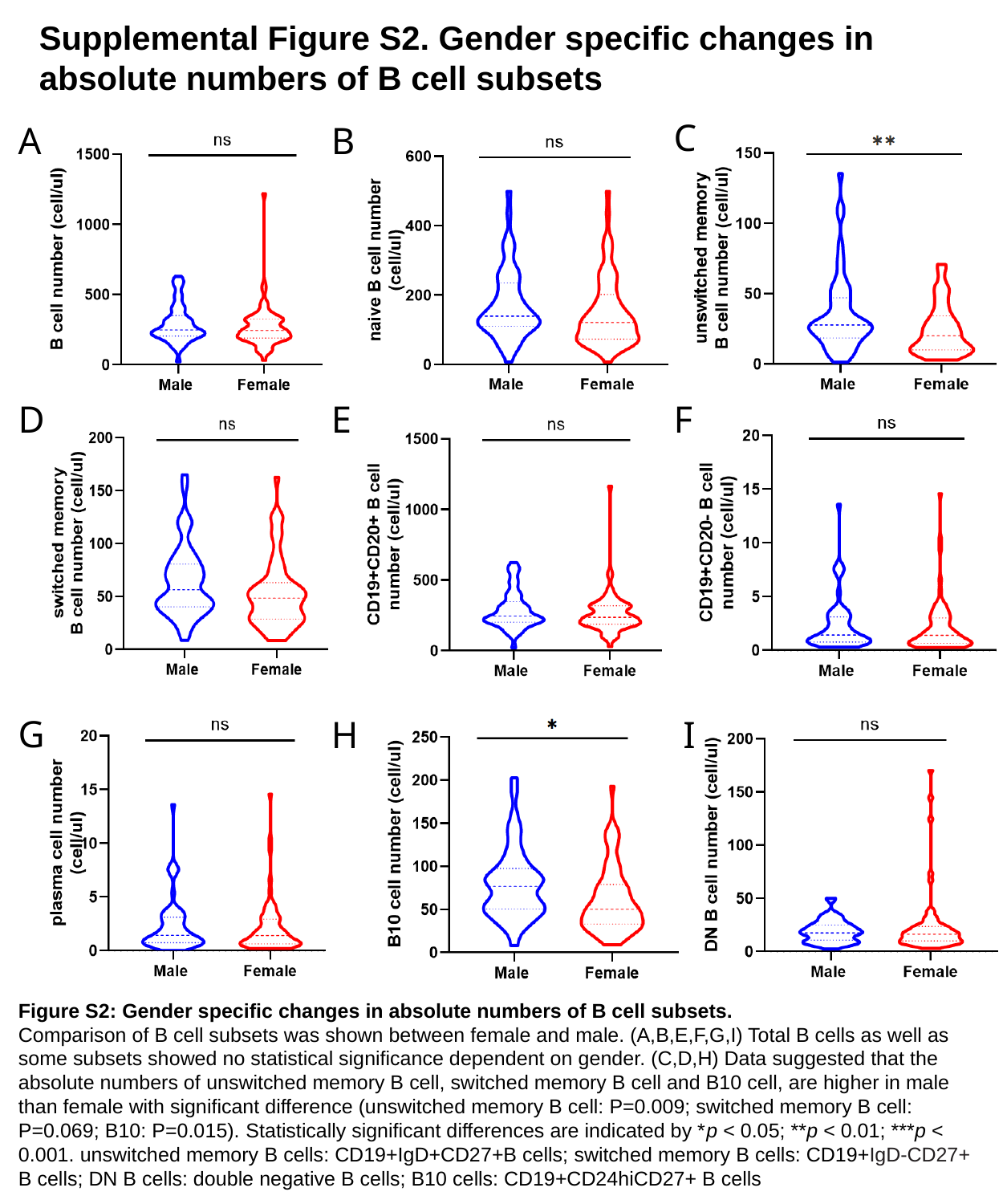

Supplemental Figure S2. Gender specific changes in absolute numbers of B cell subsets
C
A
B
F
E
D
G
H
I
Figure S2: Gender specific changes in absolute numbers of B cell subsets.
Comparison of B cell subsets was shown between female and male. (A,B,E,F,G,I) Total B cells as well as some subsets showed no statistical significance dependent on gender. (C,D,H) Data suggested that the absolute numbers of unswitched memory B cell, switched memory B cell and B10 cell, are higher in male than female with significant difference (unswitched memory B cell: P=0.009; switched memory B cell: P=0.069; B10: P=0.015). Statistically significant differences are indicated by *p < 0.05; **p < 0.01; ***p < 0.001. unswitched memory B cells: CD19+IgD+CD27+B cells; switched memory B cells: CD19+IgD-CD27+ B cells; DN B cells: double negative B cells; B10 cells: CD19+CD24hiCD27+ B cells

## Slide 4
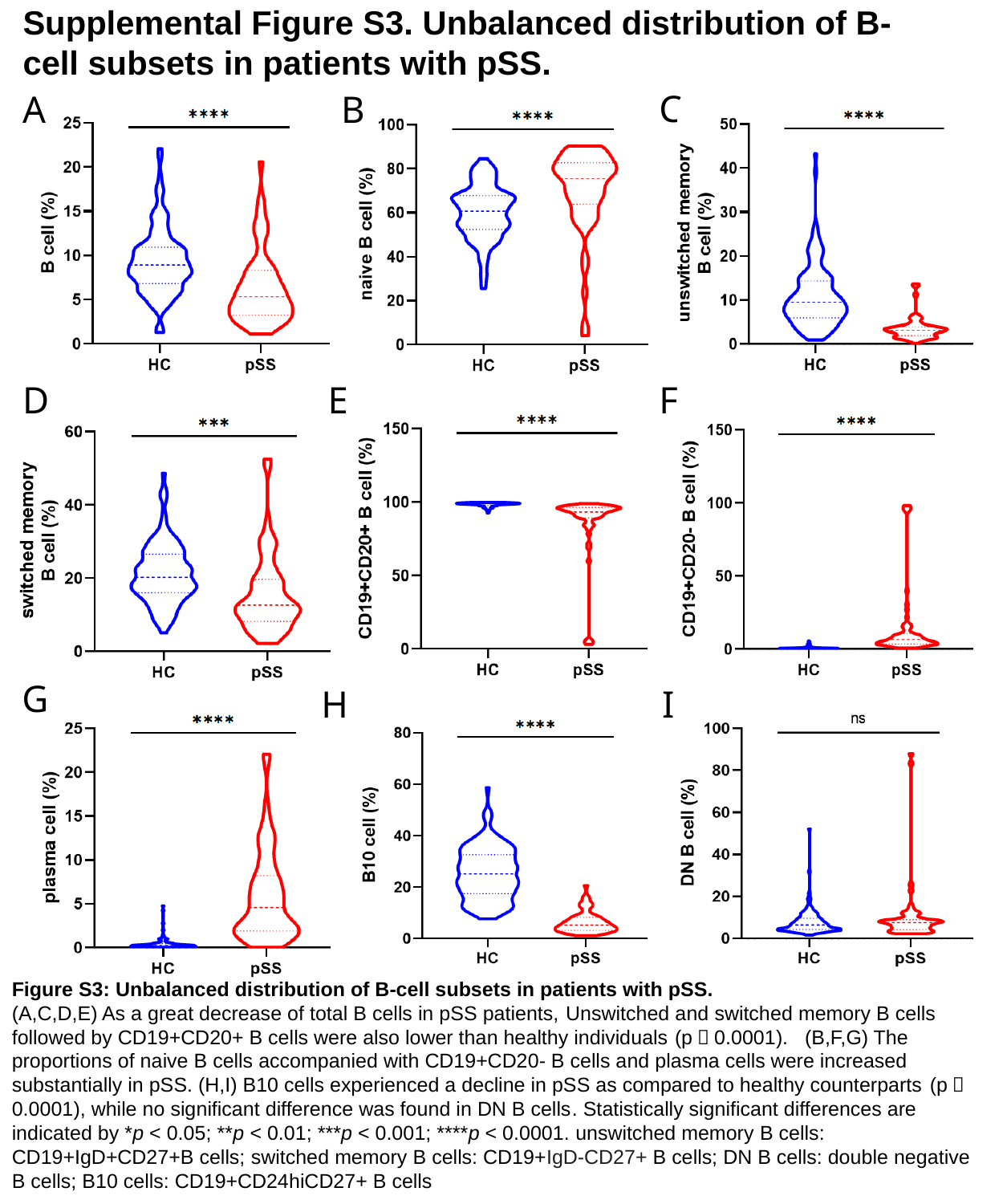

Supplemental Figure S3. Unbalanced distribution of B-cell subsets in patients with pSS.
C
A
B
D
F
E
G
H
I
Figure S3: Unbalanced distribution of B-cell subsets in patients with pSS.
(A,C,D,E) As a great decrease of total B cells in pSS patients, Unswitched and switched memory B cells followed by CD19+CD20+ B cells were also lower than healthy individuals (p＜0.0001). (B,F,G) The proportions of naive B cells accompanied with CD19+CD20- B cells and plasma cells were increased substantially in pSS. (H,I) B10 cells experienced a decline in pSS as compared to healthy counterparts (p＜0.0001), while no significant difference was found in DN B cells. Statistically significant differences are indicated by *p < 0.05; **p < 0.01; ***p < 0.001; ****p < 0.0001. unswitched memory B cells: CD19+IgD+CD27+B cells; switched memory B cells: CD19+IgD-CD27+ B cells; DN B cells: double negative B cells; B10 cells: CD19+CD24hiCD27+ B cells

## Slide 5
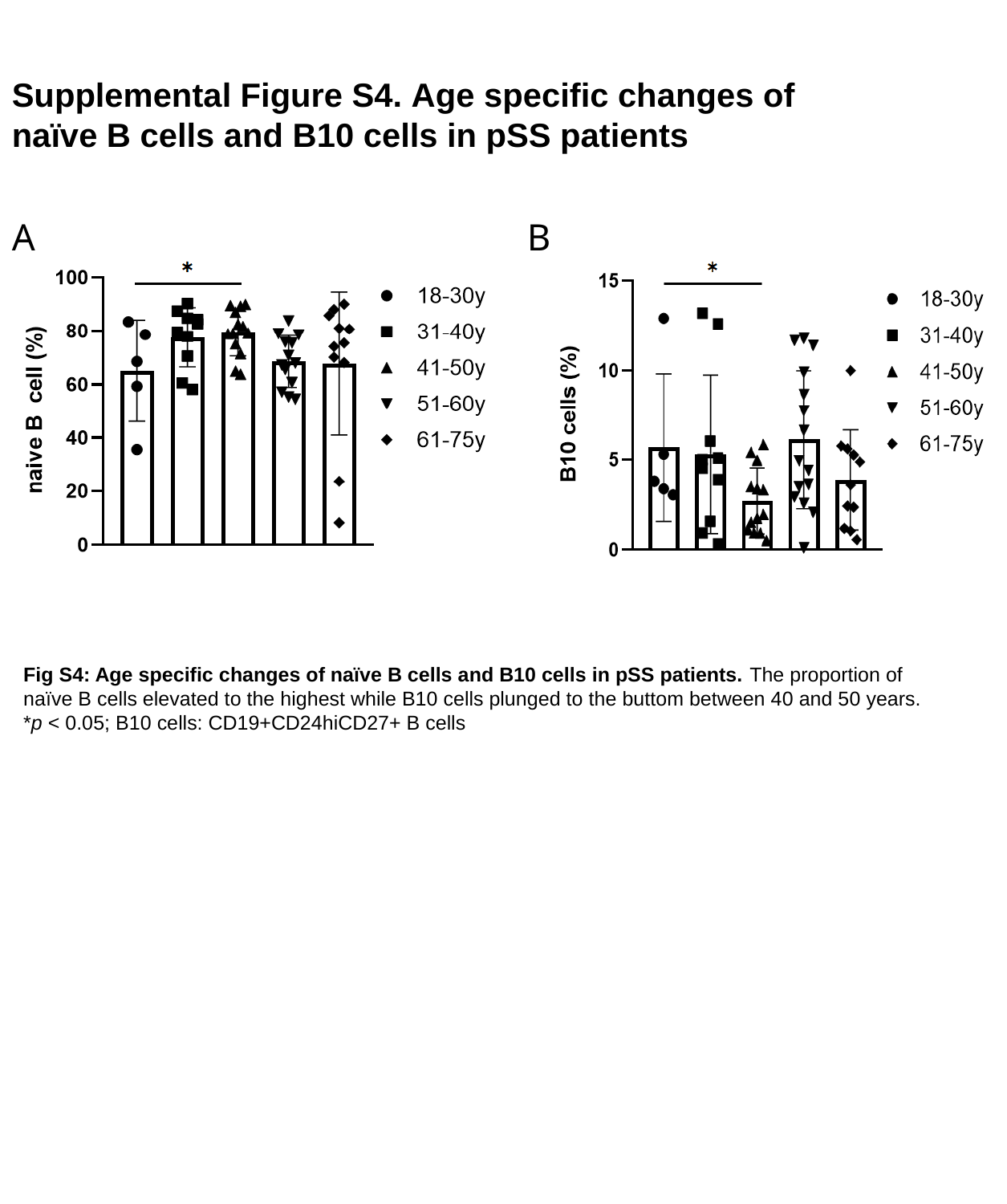

Supplemental Figure S4. Age specific changes of naïve B cells and B10 cells in pSS patients
A
B
Fig S4: Age specific changes of naïve B cells and B10 cells in pSS patients. The proportion of naïve B cells elevated to the highest while B10 cells plunged to the buttom between 40 and 50 years. *p < 0.05; B10 cells: CD19+CD24hiCD27+ B cells
